# One-Parameter Scaling Theory for DNA Extension in a Nanochannel

**DOI:** 10.1103/PhysRevLett.119.268102

**Published:** 2017-12-28

**Authors:** E. Werner, G. K. Cheong, D. Gupta, K. D. Dorfman, B. Mehlig

**Affiliations:** 1 Department of Physics, University of Gothenburg, SE-41296 Gothenburg, Sweden; 2 Department of Chemical Engineering and Materials Science, University of Minnesota–Twin Cities, 421 Washington Avenue SE, Minneapolis, Minnesota 55455, USA

## Abstract

Experiments measuring DNA extension in nanochannels are at odds with even the most basic predictions of current scaling arguments for the conformations of confined semiflexible polymers such as DNA. We show that a theory based on a weakly self-avoiding, one-dimensional “telegraph” process collapses experimental data and simulation results onto a single master curve throughout the experimentally relevant region of parameter space and explains the mechanisms at play.

As the carrier of genetic information, DNA plays a key role in biology. At the same time, recent advancements in fluorescence microscopy allow DNA to serve as a model polymer for investigating fundamental questions in polymer physics [1,2]. Nowhere is this dual importance more apparent than in the problem of DNA confinement in a nanochannel [3–5]. When the radius of gyration of the DNA molecule is larger than the channel width, it extends along the channel. This stretching lies at the heart of genome mapping in nanochannels [6]. Here the stretched DNA molecules, usually greater than 150 kilobase pairs in length, contain fluorescent markers that reveal sequence-specific information with kilobase pair resolution. This new method serves as a complement to next-generation *de novo* DNA sequencing [6–8].

Polymer confinement has been investigated for four decades, starting with the scaling arguments of Daoud and de Gennes [9]. Yet there is to date no microscopic theory explaining the experimental data of recent genome-mapping experiments [6–8,10] in narrow nanochannels. The difficulty is that the channels are too wide to apply scaling arguments derived for strong confinement [11,12], yet too narrow for the scaling arguments and theory [11,13–16] in wider channels to hold.

The challenge in developing a theory for the extension of nanoconfined DNA arises from its semiflexible nature. Semiflexible polymers are characterized by three length scales: the contour length *L*, the persistence length *ℓ_P_* quantifying the stiffness of the chain, and the effective width *w* that appears in the Onsager excluded volume [17]. For polyelectrolytes such as DNA, both the persistence length [18–21] and the effective polymer width [22] depend on electrostatic interactions. Recent experiments are often conducted in high ionic-strength buffers. In this case *ℓ_P_* is approximately 50 nm [23] while *w* is around 5 nm [24], and thus *w* ≪ *ℓ_P_*. This inequality emphasizes the intrinsic difficulty of describing DNA in a wide range of situations. DNA is considerably stiffer than typical synthetic polymers, yet the number of persistence lengths *L/ℓ_P_* in genomic DNA samples is large. Any theory for the conformational statistics of channel-confined DNA must account for both the local stiffness of the polymer and excluded-volume interactions. This is a formidable challenge. Matters are further complicated by the fact that most recent genome-mapping experiments are performed in nanochannels that are about 50 nm wide [6–8,10]. Essentially all experiments involving DNA (Fig. 1) take place in channel sizes *D* of the order of *ℓ_P_* and do not satisfy the criterion $D\gg \ell_{P}^{2}/w$ required for the scaling arguments of Ref. [9] to apply.

There is nomicroscopic theory for the extension of confined DNA for $D<\ell_{P}^{2}/w$, despite numerous attempts [28,38,39]. Scaling arguments [13,14,16] following Refs. [9,11] yield the most useful description. If *D* ≫ *ℓ_P_*, they suggest that the extension *X* scales as *X* ~ *D*^−2/3^. The problem is that the theory fails when *D* ≈ *ℓ_P_*, and as a result it proves to be a poor description of most recent DNA experiments in nanochannels. The earliest experiments [25], for instance, reported a much larger exponent *X* ~ *D*^−0.85^, and subsequent studies [32,33,40] continue to report exponents exceeding the theoretical prediction.

We take a different approach in this Letter. We show that the DNA-confinement problem for *w* ≪ *ℓ_P_* and $D\ll \ell_{P}^{2}/w$ maps to the simple one-dimensional telegraph process in Fig. 2, describing the correlated walk of a particle moving with velocity *v*_0_ along the channel axis. The velocity changes sign at rate *r*, creating hairpin configurations in the particle path. The process lasts from *t* = 0 to *t* = *T*. When the particle revisits a position it has previously visited, it incurs a penalty *ε*. We show that this model collapses experimental and simulation data for the extension throughout the experimentally relevant parameter range onto a *universal* master curve as a function of a new scaling variable *α* that measures the combined effects of confinement, local stiffness, and self-avoidance.

We start by considering narrow channels, *D* ≪ *ℓ_P_*, and later extend the arguments to channel widths up to $\ell_{P}^{2}/w$. Since we want to compute the extension of the DNA molecule along the channel axis, it suffices to consider the projection of the three-dimensional DNA configurations *x*(*s*) to the channel axis *x*. Here *s* is the contour-length coordinate; it corresponds to time *t* in the telegraph process. We decompose the probability *P*[*x*(*s*)] of observing the projected conformation *x*(*s*) as

(1) $$P[x(s)]\propto P_{\text{ideal}}[x(s)]A[x(s)].$$

The functional *P*_ideal_[*x*(*s*)] is the probability of observing the conformation *x*(*s*) in an ensemble of ideal confined polymers, disregarding self-avoidance. The functional 𝒜[*x*(*s*)] captures the effect of self-avoidance. It equals the fraction of three-dimensional polymer configurations corresponding to *x*(*s*) that contains no segments that collide with any other polymer segment.

When *D < ℓ_P_*, the local conformation statistics are determined by Odijk’s theory for narrow channels [41], while the global statistics are dominated by a random sequence of direction changes (hairpins) [42]. Neglecting self-avoidance, the separation between neighboring hairpin bends is exponentially distributed [42]. On length scales much larger than the deflection length [41] *λ* ≡ (*ℓ_P_D*^2^)^1/3^, the central-limit theorem assures that local alignment fluctuations are negligible [11]. These two facts imply that the ideal problem maps onto the one-dimensional telegraph process in Fig. 2. The correlation function of *v_x_*(*s*), the channel-axis component of the tangent vector of the ideal polymer, decays exponentially [36]:

(2) $$\langle v_{x}(s)v_{x}(0)\rangle =a^{2}\exp(-s/g).$$

The telegraph velocity has similar correlations:

(3) $$\langle v(t)v(0)\rangle =v_{0}^{2}\exp(-2rt).$$

Comparing Eqs. (2) and (3) we see that the contour parameter *s* maps to the time *t* in the telegraph process, whereupon the polymer-contour length *L* maps to the total time *T* in the telegraph model. The parameter *a* quantifies the tendency of the tangent vectors to align with the channel [31]. The parameter *g* is the global persistence length [42], characterizing the typical distance between hairpin turns. These parameters map to those of the telegraph process as follows: *a* = *v*_0_ and *g* = (2*r*)^−1^. We measured how *a* and *g* depend on the physical parameters of the full three-dimensional problem from simulations of confined ideal polymers. It turns out that it suffices to determine just two curves [Figs. 3(a) and 3(b)], since *a* and *g*/*ℓ_P_* depend on *D*/*ℓ_P_* only.

Now consider the effect of self-avoidance. In general it is very difficult to derive an expression for 𝒜[*x*(*s*)]. But for a weakly self-avoiding polymer, the problem simplifies considerably when the channel is so narrow that interactions between the polymer and the channel wall cause the three-dimensional configurations to lose correlations. We show in the Supplemental Material [36] that

(4) $$A[x(s)]\propto \exp\;\left(-\frac{\varepsilon }{2}\int dxL^{2}(x)\right)$$

if *w* ≪ *ℓ_P_*. Here ℒ(*x*)*dx* is the total amount of contour in the interval [*x; x* þ *dx*] [43]. The parameter *ε* penalizes overlaps. It is determined by the probability that two short polymer segments overlapping in one dimension collide in three dimensions [36]:

(5) $$\varepsilon =\langle \delta (y-y^{\prime })\delta (z-z^{\prime })v_{\text{ex}}\rangle /\ell^{2}.$$

The average is over the conformations of the confined *ideal* polymer, and *y* and *z* are the transverse channel coordinates of a short polymer segment of length *ℓ*. Primed coordinates belong to a second, independent segment, and *v*_ex_ is the excluded volume. The excluded volume depends on the segment orientation. If *ℓ* ≫ *w*, we have *v*_ex_ = 2*wℓ*^2^ sin *θ*, where *θ* is the angle between the two segments [17]. Figure 3(c) shows how *ε* depends on *D*/*ℓ_P_*, obtained by evaluating the average in Eq. (5) from three-dimensional simulations of confined ideal polymers [36]. A single curve is sufficient to determine how *ε* depends on the physical parameters, because *εD*^2^/*w* is a function of *D*/*ℓ_P_* only (see Supplemental Material [36]).

In the telegraph model self-avoidance is incorporated in the same way. Here, ℒ has units of (time)/(position). Equation (4) then shows that *ε* has units of (position)/(time)^2^. Since *r* has units of (time)^−1^, and *v*_0_ of (position)/(time), the only dimensionless combination of *ε*, *r*, and *v*_0_ is

(6) $$\alpha \equiv \varepsilon /(2v_{0}r).$$

In the limit of large *T*, only *α* can have physical significance. Invoking our mapping between telegraph model and polymer problem, we conclude that *α* is given by

(7) α=εg/a.

This parameter measures the expected number of overlaps between the two strands of a hairpin of length *g*.

Equation (7) has two important consequences. First, Eq. (7) allows us to generalize the mapping to all channel widths up to $\ell_{P}^{2}/w$. To show this, consider first the ideal part. Strictly speaking, the simple picture outlined above breaks down when *D* ~ *ℓ_P_* because the typical hairpin length *g* becomes of the same order as *ℓ_P_*. But consider how *α* changes as *D* approaches *ℓ_P_* from below. For *w* ≪ *ℓ_P_*, the parameter *α* decreases below unity before *g* = *ℓ_P_* is reached, and for small *α* the precise nature of the local conformations is irrelevant. All that matters is that the ideal part is a diffusion process with exponentially decaying correlations of *v_x_*(*t*). Similarly, the local probability of collision is still (*ε*/2)ℒ^2^(*x*)*dx*, because each segment pair collides independently. The latter assumption eventually breaks down at $D\approx \ell_{P}^{2}/w$ since the transversal segment coordinates become correlated. But up to this point Eq. (4) is valid, as is Eq. (5).

Second, observables that are dimensionless in the telegraph model can only depend on *α*, Eq. (7), in the limit of large *L*. This combination *α* is plotted in Fig. 3(d). It turns out that *αD*^2^/(*ℓ_P_w*) depends only on *D*/*ℓ_P_* [36]. Now consider the average extension *X* and the variance about that average *σ*^2^. In the telegraph model these quantities have units of (position) and (position)^2^, and for large values of *L* they must be proportional to *L*. We therefore conclude that the data must collapse as

(8) $$X/(La)=f_{X}(\alpha )\;\;\;\text{and}\;\;\;\sigma^{2}/({\mathrm{Lga}}^{2})=f_{\sigma }(\alpha ).$$

Here *f_X_* and *f_σ_* are *universal* scaling functions that depend only on *α*. We can numerically compute the form of these functions simply by simulating the telegraph model [36].

We have compared our theory to direct numerical simulations (DNS) of three-dimensional, confined, self-avoiding wormlike chains [39] using the PERM algorithm [44,45]. Figures 4(a) and 4(b) show that our theory quantitatively captures the DNS results for all values of *w*/*ℓ_P_* tested [36], up to *w*/*ℓ_P_* = 0.4. This agreement is remarkable, as the theory assumes weak self-avoidance, *w* ≪ *ℓ_P_*.

Figures 4(c) and 4(d) show the comparison between the results of the experiments summarized in Fig. 1 and our theory. The theory not only collapses the experimental data, but provides good quantitative agreement, in particular with the most recent experiments [10,33,34]. There is some scatter of the experimental data around the theoretical curve, but this is expected because the nanofluidic experiments are quite difficult to control.

In the limit *α* ≪ 1 our theory allows us to map the problem to an uncorrelated weakly self-avoiding diffusion process [43,47]. This follows from the fact that the correlation function in the telegraph model, Eq. (3), decays to zero before the next collision occurs, for *α* ≪ 1. As a result, the ideal random walk is simply diffusive, with diffusion constant $D=v_{0}^{2}/(2r)$. This has two consequences.

First, for *α* ≪ 1 observables depend on *v*_0_ and *r* only through the combination 𝒟. Since the extension is linear in *L* for large *L*, we deduce that *X*/*L* can only depend on *ε* and 𝒟 in this limit. Since 𝒟 has units of (position)^2^/(time) while *ε* has units of (position)/(time)^2^ in the telegraph model, we see that the only possible combination is *X*/*L* ∝ (*ε*𝒟)^1/3^. This gives *X*/(*La*)=*f_X_*(*α*)∝*α*^1/3^, explaining the power law in Figs. 4(a) and 4(c). For the variance we conclude that *σ*^2^/*L* ∝ 𝒟, independent of *ε* [Figs. 4(b) and 4(d)]. Alternatively, we can deduce these scalings by a mean-field argument, indicating that fluctuations of ℒ are negligible when *α* ≪ 1. Assuming that ℒ ~ *T*/*X*, we find for the extension distribution $P(X)\sim \exp[-rX^{2}/(2v_{0}^{2}T)-(\varepsilon /2)T^{2}/X]$, whereupon *d* log *P*/*dX* = 0 yields *X*/(*v*_0_*T*) ~ *α*^1/3^. For the variance we obtain $\sigma^{2}r/(v_{0}^{2}T)\sim \alpha^{0}$.

Second, we can use the exact mathematical results derived in Ref. [47] to deduce the prefactors:

(9a) $$f_{X}(\alpha )=c_{X}\alpha^{1/3}\;\;\;(1.104\leq c_{X}\leq 1.124),$$

(9b) $$f_{\sigma }(\alpha )=c_{\sigma }\;\;\;(0.72\leq c_{\sigma }\leq 0.87),$$

as *α* → 0. The constraints for *c_X_* and *c_σ_* are rigorously proven mathematical bounds [47].

Now consider the limit of large *α*. The extension *X* tends to *La* in this limit [11], since the frequency of hairpins tends to zero. The variance decays as *σ*^2^ ~ *α*^−3^, as Fig. 4 shows. To deduce this power law, we estimate the variance of the strongly extended polymer as (number of hairpins)× (hairpin extension)^2^. To determine the number of hairpins, note that the expected number of collisions for a hairpin of contour length *h* is ~*αh*/*g*. The resulting hairpin is therefore likely to survive the collision check only if *h* is of the order *g*/*α* or smaller. This requires a second switch of direction within the length *g*/*α*. This occurs with probability (*g*/*α*)/*g* = *α*^−1^, so that the number of hairpins is (*L*/*g*)*α*^−1^. To obtain the hairpin extension we multiply its contour length *h* ~ *g*/*α* by its alignment *a*, so that the typical hairpin extension becomes ~*ga*/*α*. Therefore, *σ*^2^ ∝ (*L*/*g*)*α*^−1^(*ga*/*α*)^2^ = *Lga*^2^*α*^−3^ for large *α*.

For very large values of *α*, the theory fails [Fig. 4(b)] because hairpins are so rare that alignment fluctuations (not included in the telegraph model) dominate the variance [11]. This correction is taken into account simply by adding the variance in the extreme Odijk limit [48]:

(10) $$\sigma^{2}=d_{\sigma }({\mathrm{Lga}}^{2})\alpha^{-3}+\sigma_{\text{Odijk}}^{2}\;\;\;\text{as}\;\alpha \to \infty .$$

Here *d_σ_* is a universal constant. By fitting the solid line in Fig. 4(b) for *α* > 10, we find *d_σ_* ≈ 0.09. We observe excellent agreement between this refined theory and the simulation data for *ℓ_P_*/*w* = 12. For the stiffer polymers (*ℓ_P_*/*w* = 36), still longer contour lengths are required to reach the large-*L* limit and to reduce the statistical error.

Finally, we show that our theory contains scaling laws derived earlier as particular asymptotic limits. In very narrow channels, *a* ≈ 1 and 〈sin *θ*〉 ≈ (*D*/*ℓ_P_*)^1/3^. Using these approximations in Eqs. (5) and (6) gives

(11) $$\alpha =\mathrm{Cgw}{\left(D^{5}\ell_{P}\right)}^{-1/3}=C\xi \;\;\;(D\ll \ell_{P}),$$

where *C* ≈ 1.95 is a constant [36]. The parameter *ξ* appears in Odijk’s scaling theory [11] and the extension scales as *X* ~ *ξ*^1/3^ [11] in this special limit. In wide channels, for $\ell_{P}\ll D\ll \ell_{P}^{2}/w$, we have that $a=1/\sqrt{3}$ and *g* ≈ *ℓ_P_*. Using diffusion approximations for the distribution of the polymer in the channel [36] gives

(12) $$\alpha =9\sqrt{3}\pi w\ell_{P}/(8D^{2})\ll 1\;\;\;(\ell_{P}\ll D\ll \ell_{P}^{2}/w).$$

This is the result of Ref. [15], implying the same scaling *X* ~ *D*^−2/3^ that Odijk’s scaling arguments [11] predict in this asymptotic limit. At first glance it is perhaps surprising that the two distinct limits Eqs. (11) and (12) are described by the same random-walk process. After all, the three-dimensional polymer conformations are entirely different in the two regimes, described by invoking deflection segments, hairpins, and blobs. Our universal theory, by contrast, rests on the fact that the macroscopic statistics of a weakly interacting random walk do not depend on the microscopic details of the process [49].

We can also conclude that the DNA experiments shown in Fig. 1 cannot obey the scalings *X* ~ *D*^−2/3^ or *X* ~ *ξ*^1/3^ because the experiments do not satisfy the strong inequalities *D* ≫ *ℓ_P_* or *D* ≪ *ℓ_P_* (see Fig. S-5 in the Supplemental Material [36]), and therefore do not reach the asymptotic limits required for these power laws to emerge. Our theory shows, and Fig. 4(a) confirms, that *X* ~ *α*^1/3^ for small values of *α*. But the parameter *α* depends upon *D* and *ℓ_P_* in an intricate way via Eq. (7), in general not in a power-law fashion.

In summary, we have shown that DNA confinement in nanochannels can be modeled by a telegraph process, collapsing all of the data in terms of a scaling variable *α*. Our theory brings to the fore universal properties of confined polymers in a good solvent in a way that is obscured by the prevailing scaling theories [9,11,12,14,15,41]. The distinction between deflection segments, hairpins, and blobs, which leads to the need to define separate regimes, is not necessary. Rather, the statistics of the confined polymer chain for $D≾\ell_{P}^{2}/w$ adopt a universal behavior at sufficiently long length scales, independent of the details of the microscopic model.

## Supplementary Material

SI

## Figures and Tables

**FIG. 1:**
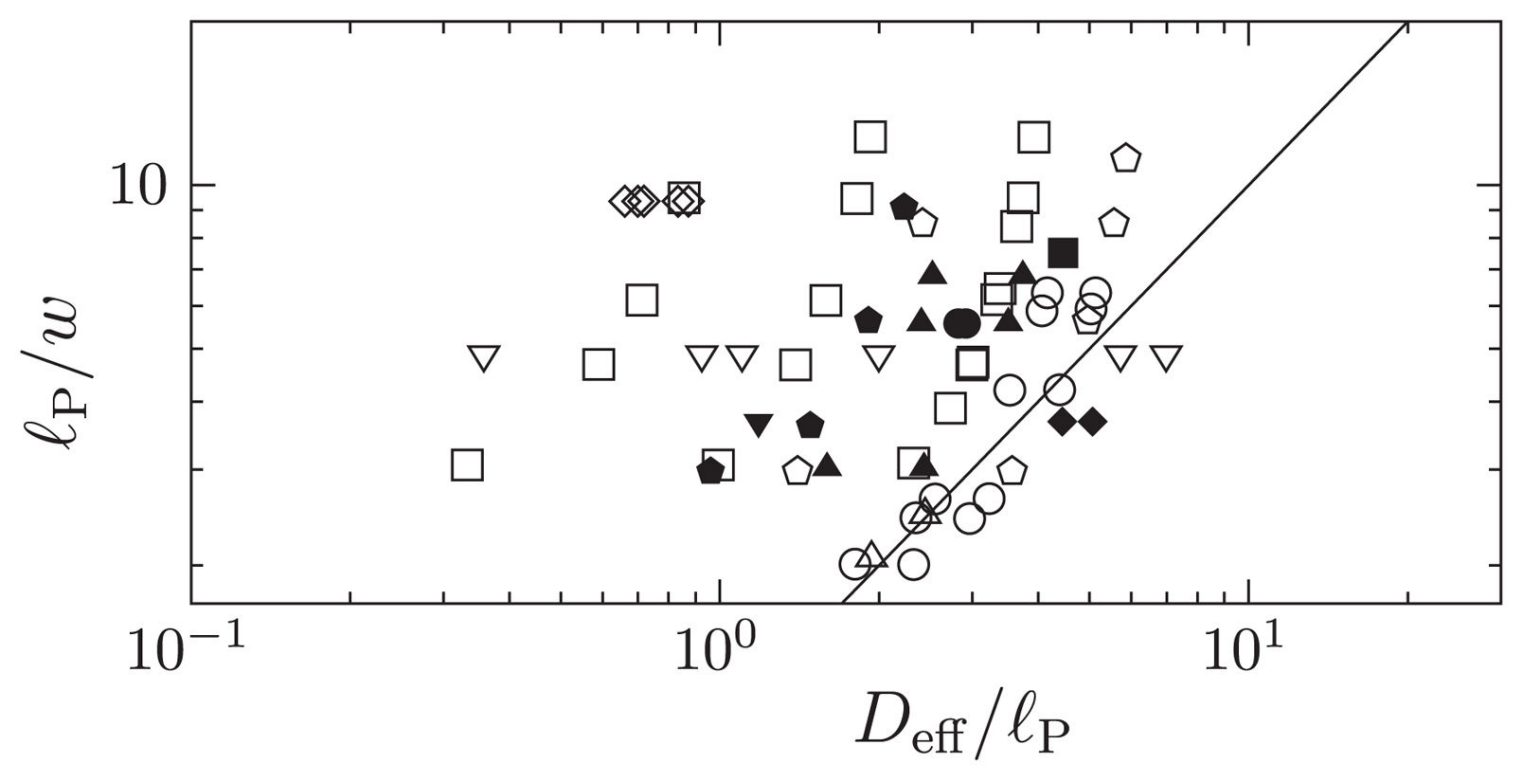
Parameters for experiments on nanoconfined DNA: inverted triangle [25], square [26], filled square [27], circle [28], filled circle [29], triangle [30], filled triangle [31], inverted filled triangle [32], diamond [10], filled diamond [33], pentagon [34], and filled pentagon [35]. For experiments using funnels [29,31,33] only maximum and minimum channel widths are indicated. The methods for selecting the data sets, and for computing the “effective channel width” *D*_eff_, *ℓ_P_*, and *w* from the experimental parameters, are described in the Supplemental Material [36]. Solid line shows $D_{\text{eff}}=\ell_{P}^{2}/w$.

**FIG. 2:**
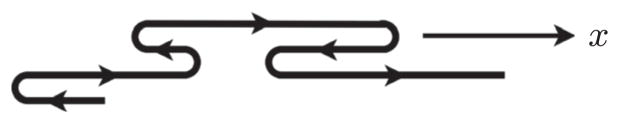
Illustration of the telegraph process along the channel axis (*x* axis). The walk is one dimensional, but for clarity it is expanded vertically, to show the changes in direction that create hairpin configurations of the confined DNA molecule.

**FIG. 3:**
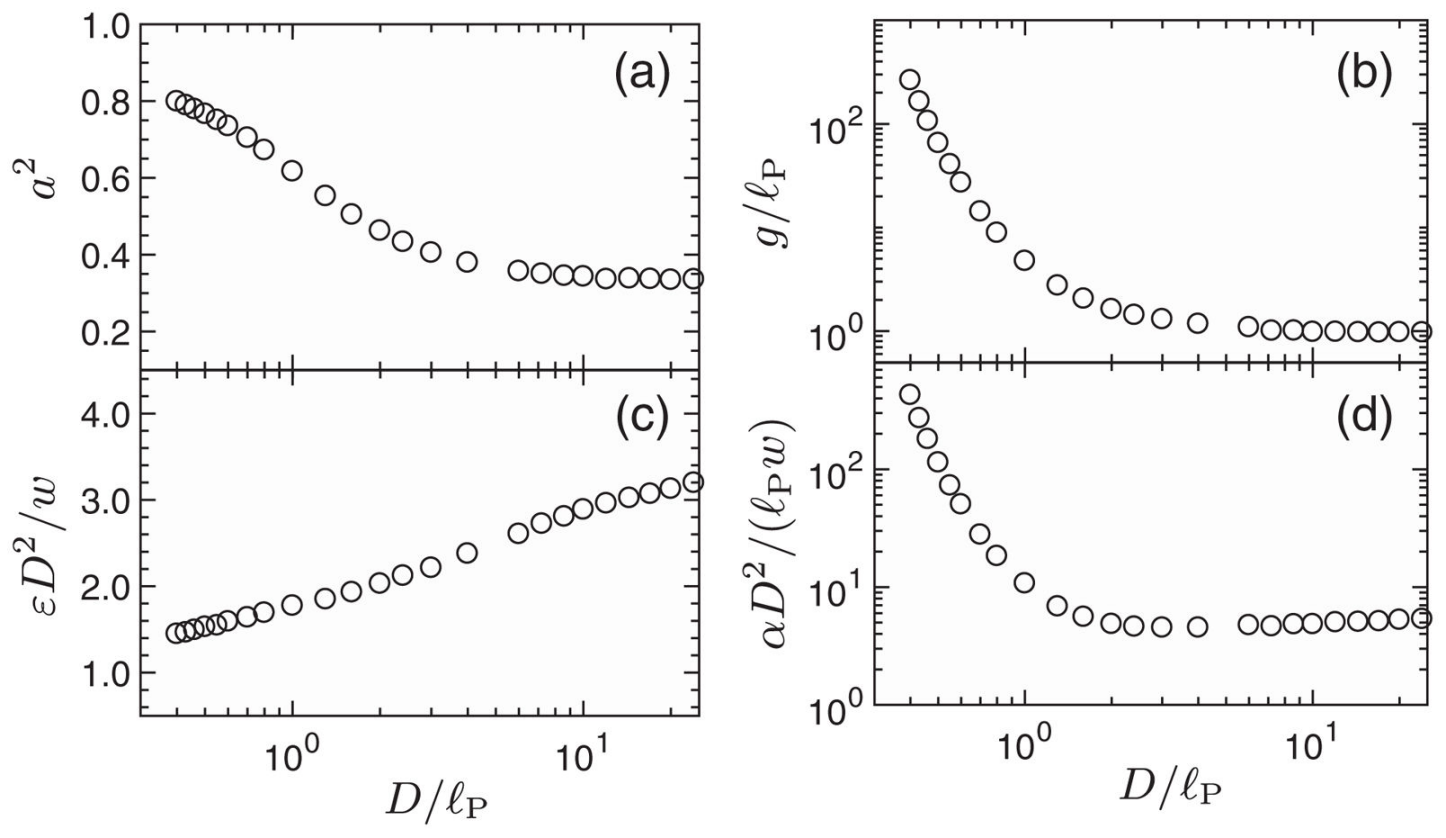
Results from ideal-polymer simulations [36] showing how *a* = *v*_0_, *g* = 1/(2*r*), *ε*, and *α* depend on *D*/*ℓ_P_*.

**FIG. 4:**
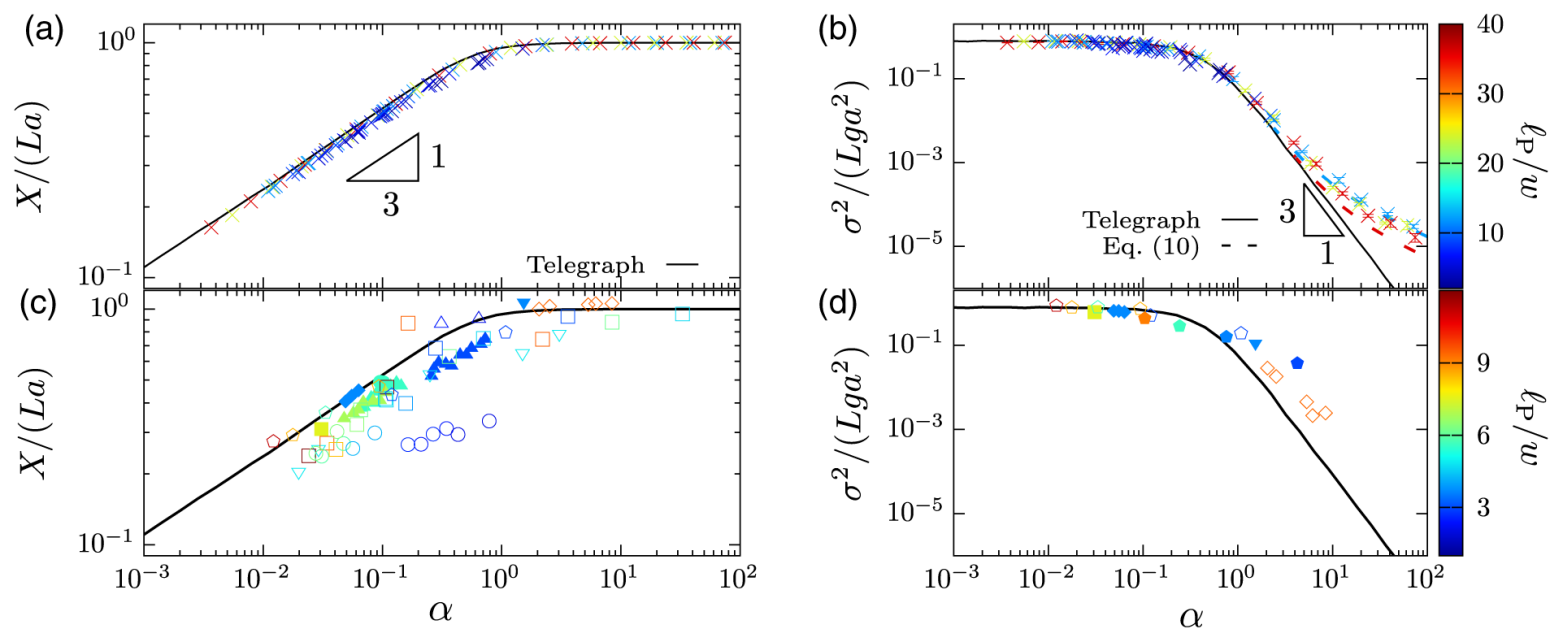
One-parameter scaling of the mean extension *X* and the extension variance *σ*^2^. Comparison of one-parameter theory (solid black lines) to results of three-dimensional direct numerical simulations (DNS) (a), (b) and experiments (c), (d). DNS: crosses. The DNS method [39,46] is described in the Supplemental Material [36]. Experiments (same as in Fig. 1): ▽ [25], square [26], filled square [27], circle [28], filled circle [29], triangle [30], filled triangle [31], inverted filled triangle [32], diamond [10], filled diamond [33], pentagon [34], and filled pentagon [35]. The details of these experiments and the selection of experimental data sets are described in the Supplemental Material [36]. In addition, the predicted scalings for the mean extension, *X* ~ *α*^1/3^, and for the extension variance, *σ*^2^ ~ *α*^−3^, are indicated. The color bar shows the range of *ℓ_P_*/*w* for DNS (top) and experiments (bottom). The dashed lines in (b) show theoretical predictions from Eq. (10) for *ℓ_P_*/*w* = 12 (dashed blue line) and 36 (dashed red line). See Fig. S-2 in Supplemental Material [36] for the telegraph-model results as a function of channel width *D*.
