# Supplementary material for: One-Parameter Scaling Theory for DNA Extension in a Nanochannel
Source: Phys Rev Lett. Author manuscript; Available in PMC 2018 Jan 16. (PMC5769985; doi:10.1103/PhysRevLett.119.268102)
Supplement: SI [file NIHMS931023-supplement-SI.pdf]

Supplemental Material for "One-parameter scaling theory for DNA extension in a nanochannel"

E. Werner,[1] G. K. Cheong,[2] D. Gupta,[2] K. D. Dorfman,[2,a] and B. Mehlig[1,b]

[1] Department of Physics, University of Gothenburg, SE-41296 Gothenburg, Sweden

[2] Department of Chemical Engineering and Materials Science, University of Minnesota – Twin Cities, 421 Washington Avenue SE, Minneapolis, Minnesota 55455, USA

[a] Email: dorfman@umn.edu

[b] Email: bernhard.mehlig@physics.gu.se

Contents

S-1 Telegraph process **2**

S-1.1 Derivation of Eq. (4) in the main text 2

S-1.2 Derivation of Eq. (5) in the main text 5

S-1.3 Asymptotic limits for large and small α 6

S-1.4 Computer simulations of telegraph model 7

S-2 Ideal-polymer simulations **9**

S-2.1 Ideal tangent correlations. Determination of g and a in terms of D and $\ell_{\rm P}$. 10

S-2.2 Determination of the parameter ε in terms of $D, w, \ell_{\rm P}$ 11

S-2.3 Known asymptotes of g, a and p for small and large $D/\ell_{\rm P}$ 12

S-3 Direct numerical simulations of the three-dimensional self-avoiding polymer in confinement **13**

S-4 Summary of experimental data analyzed in main text **17**

S-4.1 Computation of contour length, persistence length and effective polymer width 17

S-4.2 Tabulation of experimental data 17

S-4.3 Experimental parameters in relation to asymptotic scaling regimes 24

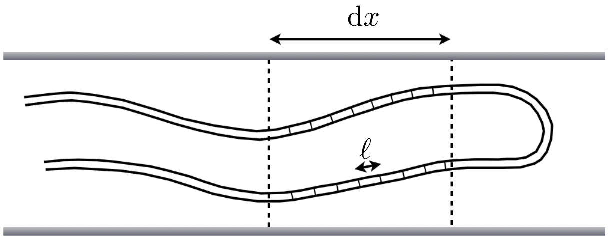

Figure S-1: Illustration of two overlapping strands of a DNA molecule confined to a narrow channel. Discretization of the problem to derive the telegraph model, $w \ll \ell \ll \mathrm{d}x$. Here w is the effective width of the DNA molecule, ℓ is the length of a short DNA segment, and $\mathrm{d}x$ is the discretization in the channel direction.

S-1 Telegraph process

Eqs. (1) to (7) in the main text show how the problem of computing the extension of a DNA molecule in a nanochannel is mapped to a one-dimensional telegraph process. In this Section we give all details necessary to derive this mapping, to evaluate asymptotic limiting cases, and to simulate the process.

S-1.1 Derivation of Eq. (4) in the main text

Consider a weakly self-avoiding polymer ($w \ll D$) in a narrow channel ($D \ll \ell_{\mathrm{P}}$). We divide the channel into slices of width $\mathrm{d}x$ and assume that $\mathrm{d}x \gg \lambda$, where $\lambda \equiv (\ell_{\mathrm{P}} D^2)^{1/3}$ is the Odijk deflection length,[1] so that we can neglect fluctuations in the contour length and alignment of a hairpin strand. We also assume that $\mathrm{d}x$ is small enough so that $p_{\mathrm{coll}}(x)\mathrm{d}x \ll 1$. Here $p_{\mathrm{coll}}(x)\mathrm{d}x$ is the probability that one or more collisions occur in the channel segment between x and $x + \mathrm{d}x$. This assumption assures that we can neglect higher-order terms in a Taylor expansion below. Provided that $w \ll D$ it is possible to satisfy both inequalities simultaneously for all configurations $x(t)$ that have non-negligible weight in the ensemble. The probability $\mathscr{A}[x(t)]$ that the polymer configuration is free of overlaps is given by

$$\mathscr{A}[x(t)] = \prod_{\text{all slices}} \big(1 - p_{\mathrm{coll}}(x)\,\mathrm{d}x\big) = \exp\Big\{ \sum_{\text{all slices}} \log\big[1 - p_{\mathrm{coll}}(x)\mathrm{d}x\big] \Big\}\,.$$

Since $p_{\rm coll}(x){\rm d}x \ll 1$ this expression equals

$$= \exp\big\{ -\sum_{\text{all slices}} p_{\rm coll}(x){\rm d}x \big\} = \exp\big\{ -\int {\rm d}x\; p_{\rm coll}(x) \big\}\,. \tag{S-1}$$

To evaluate the integral, we consider the two overlapping strands as shown in Fig. S-1. What is the probability that they collide in the interval between x and $x + {\rm d}x$? To answer this question we divide each strand into many short segments of length ℓ. We must assume that $w \ll \ell$, so that collision checks between neighboring segments are independent. In the interval from x to $x + {\rm d}x$ there are $(L_{\rm s}/\ell)^2$ segments pairs to check, $(L_{\rm s}/\ell)$ on each strand. Here $L_{\rm s}$ is the average strand length in the interval. Assume that the interval contains $N_{\rm s}$ strands. Then the probability that there is at least one collision in the interval from x to $x + {\rm d}x$ is

$$p_{\rm coll}(x)\,{\rm d}x = p\,\Big(\frac{L_{\rm s}}{\ell}\Big)^2 N_{\rm s}(N_{\rm s}-1)/2\,. \tag{S-2}$$

Here p is the probability that two randomly chosen short segments collide. Now we introduce the 'local time'[2] $\mathscr{L}(x)$, where $\mathscr{L}(x){\rm d}x$ is defined as the total contour length of the configuration $[x(t)]$ occupying $[x, x + {\rm d}x]$. It follows from this definition that $\mathscr{L}$ is in fact a density, it has units time/position. In terms of $\mathscr{L}(x)$ Eq. (S-2) becomes:

$$p_{\rm coll}(x)\,{\rm d}x = \frac{p\,{\rm d}x}{2\ell^2}\left[\mathscr{L}^2(x)\,{\rm d}x - \mathscr{L}(x)L_{\rm s}\right] = \frac{\varepsilon}{2}\left[\mathscr{L}^2(x)\,{\rm d}x - \mathscr{L}(x)L_{\rm s}\right], \tag{S-3}$$

where we have introduced the 'penalty' parameter

$$\varepsilon \equiv \frac{p\,{\rm d}x}{\ell^2}\,. \tag{S-4}$$

It has units of position/time^2. Upon integration over x the first term gives Eq. (4) in the main text:

$$\mathscr{A}[x(t)] = \mathscr{A}_0 \exp\big\{ -\tfrac{\varepsilon}{2}\int {\rm d}x\, \mathscr{L}^2(x) \big\}\,. \tag{S-5}$$

The second term gives the normalization factor $\mathscr{A}_0$. We evaluate it using $L_\mathrm{s} = \mathrm{d}x/a$:

$$\mathscr{A}_0 = \exp\big\{\tfrac{\varepsilon L}{2a}\big\}\,. \tag{S-6}$$

Here L is the contour length of the polymer. This concludes the derivation of Eq. (4) in the main text for $D \ll \ell_\mathrm{P}$. Our derivation assumes that different channel slices can be treated independently, and that segment pairs within a slice are independent. The first assumption is satisfied in narrow channels ($D \ll \ell_\mathrm{P}$) since $\mathrm{d}x \gg \lambda$. The second assumption holds since $p_\mathrm{coll}(x)\mathrm{d}x \ll 1$ because this condition ensures that the result of a collision check for a pair of segments is independent of the result of checking any other segment pair.

Now consider what happens when D increases. Fig 3(b) in the main text shows that the global persistence length decreases as D grows, and the picture underlying the derivation outlined above breaks down when $g = \ell_\mathrm{P}$ because the notion of well aligned strands no longer applies in this case. This is, however, of little consequence because the universal scaling parameter α decreases below unity before $g = \ell_\mathrm{P}$ is reached. For small values of α, the correlations of the tangent vectors decay before a collision occurs. For large-contour length separations the tangent vectors thus perform an uncorrelated random walk with diffusion constant $\mathscr{D} = a^2 g = v_0^2/(2r)$. Local conformations are no longer described by hairpins, but the precise nature of the local conformations is irrelevant in this limit, because the macroscopic conformation statistics of a self-avoiding polymer do not depend on the microscopic model.[3] The ideal probability is simply Gaussian at contour-length scales above the global persistence length g. The telegraph process can still be used because it gives precisely this ideal distribution in the limit of $\alpha \ll 1$.

Now consider the effect of self-avoidance. As $g \to \ell_\mathrm{P}$ the local conformations no longer resemble hairpins, so that the number of hairpin strands ceases to be well defined. But Eq. (S-5) remains valid, because this equation simply expresses the fact that the probability of a collision in a channel slice is proportional to the number of segment pairs in that slice. Eventually, however, Eq. (S-5) must break down, when transversal segment coordinates become correlated. This occurs for $D \gg \ell_\mathrm{P}^2/w$.

S-1.2 Derivation of Eq. (5) in the main text

Recall that p denotes the probability that two short segments of length ℓ collide, given that they occupy the same channel slice of width $\mathrm{d}x$. Denote the positions of the two segments by $\boldsymbol{x} = (x, y, z)^\mathsf{T}$ and $\boldsymbol{x}' = (x', y', z')^\mathsf{T}$, and the tangent vectors by $\boldsymbol{v}$ and $\boldsymbol{v}'$. The collision probability p can be written as

$$p = \langle \chi(\boldsymbol{x} - \boldsymbol{x}', \boldsymbol{v}, \boldsymbol{v}') \rangle \,. \tag{S-7}$$

Here $\langle \ldots \rangle$ is an average over $\boldsymbol{x}, \boldsymbol{v}$ and $\boldsymbol{x}', \boldsymbol{v}'$, distributed with the independent probabilities $p(\boldsymbol{x}, \boldsymbol{v})$ and $p(\boldsymbol{x}', \boldsymbol{v}')$ of the two segments in the interior of the polymer.[4] The indicator function $\chi(\boldsymbol{x} - \boldsymbol{x}', \boldsymbol{v}, \boldsymbol{v}')$ equals unity when the two segments collide, and zero otherwise. Since $D \gg \ell$, the probability density $p(\boldsymbol{x}, \boldsymbol{v})$ varies little as $\boldsymbol{x}$ changes over the length of the segment. We can therefore write

$$\chi = \delta(\boldsymbol{x} - \boldsymbol{x}') v_{\mathrm{ex}}(\boldsymbol{v}, \boldsymbol{v}') \,, \tag{S-8}$$

where $v_{\mathrm{ex}}(\boldsymbol{v}, \boldsymbol{v}')$ is the volume of the region surrounding a segment with tangent $\boldsymbol{v}$ that is excluded (on average) to other segments that have tangent vector $\boldsymbol{v}'$. For $\ell \gg w$ we can use Onsager's result[5] for the excluded volume:

$$v_{\mathrm{ex}}(\boldsymbol{v}, \boldsymbol{v}') = 2\ell^2 w \sin\theta(\boldsymbol{v}, \boldsymbol{v}') \,. \tag{S-9}$$

Here $\theta(\boldsymbol{v}, \boldsymbol{v}')$ is the angle between the two segments with tangents $\boldsymbol{v}$ and $\boldsymbol{v}'$. Finally, we note that the distributions of x and x' are simply uniform in the slice of width $\mathrm{d}x$. This yields

$$p = \langle \delta(y - y')\delta(z - z') v_{\mathrm{ex}}(\boldsymbol{v}, \boldsymbol{v}') \rangle / \mathrm{d}x, \tag{S-10}$$

or, using the definition (S-4) of ε,

$$\varepsilon = \langle \delta(y - y')\delta(z - z') v_{\mathrm{ex}}(\boldsymbol{v}, \boldsymbol{v}') \rangle / \ell^2 \,. \tag{S-11}$$

This is Eq. (5) in the main text. The derivation did not make any assumptions about the channel width, D. This means that (S-11) is valid throughout the regime of validity of (S-5).

S-1.3 Asymptotic limits for large and small α

Eqs. (11) and (12) in the main text describe asymptotic limits of the telegraph process. Here we give additional details for these two asymptotic limits.

First when $\ell_p \ll D \ll \ell_{\rm P}^2/w$ then the correlation function is the same as that of the unconfined DNA, wherein $a = 1/\sqrt{3}$ and $g = \ell_{\rm P}$.[6] To calculate ε, we use a diffusion approximation[4] for $p(\boldsymbol{x}_\perp, \boldsymbol{v})$, the joint distribution of $\boldsymbol{x}_\perp = (y, z)^{\sf T}$ and $\boldsymbol{v}$:

$$p(\boldsymbol{x}_\perp, \boldsymbol{v}) = \frac{1}{4\pi}\frac{4}{D^2}\sin^2(\pi y/D)\sin^2(\pi z/D)\,. \tag{S-12}$$

The factor of $(4\pi)^{-1}$ comes from the fact that angular orientations are isotropic in this limit. Using these results in Eq. (S-11) gives

$$\varepsilon = \frac{9\pi}{8}\frac{w}{D^2}\,. \tag{S-13}$$

We can recover the results of Werner and Mehlig[6] by setting the discretization scale ℓ to $2\ell_{\rm P}$, whereupon we obtain Eq. (3) of Werner and Mehlig:[6]

$$\alpha = \frac{9\sqrt{3}\pi}{8}\frac{w\ell_{\rm P}}{D^2}\,. \tag{S-14}$$

Second when $D \ll \ell_{\rm P}$, the prefactor for the correlation function approaches $a = 1$. While there is no diffusion approximation for the Odijk regime, we know from dimensional analysis that the probability density of $\boldsymbol{x}_\perp$ must be of the form $p(\boldsymbol{x}_\perp) = D^{-2}h(y/D)h(z/D)$, and $\langle \sin(\theta)\rangle \approx (D/\ell_{\rm P})^{1/3}$, see Ref. 7. As a result we find from Eq. (S-11)

$$\varepsilon \approx \frac{Cw}{D^{5/3}\ell_{\rm P}^{1/3}} \tag{S-15}$$

with an unknown numerical prefactor C. Fitting the factor C in Eq. (S-15) to the ideal-

polymer simulations in Fig. S-4(c) gives $C \approx 1.95$. For α we find the expression:

$$\alpha \approx C \frac{wg}{D^{5/3}\ell_{\mathrm{P}}^{1/3}} \,, \tag{S-16}$$

where the right-hand side is proportional to the parameter ξ defined by Odijk.[7] We emphasize that our telegraph theory does not contain any unknown factors. The numerical constant C occurs in the relation between our exact theory and the parameter ξ in the asymptotic limit $D \ll \ell_{\mathrm{P}}$.

S-1.4 Computer simulations of telegraph model

The solid lines in Fig. 4 in the main text were obtained by simulating a discretized version of the weakly self-avoiding telegraph model described in the main text, with $v_0 = a = 1$. We discretize the telegraph process into segments of length ℓ as follows. If step number $j-1$ is in the positive (negative) direction, then step number j is taken in the opposite direction with probability $p_{\mathrm{switch}} = r\ell$, and in the same direction with probability $1 - p_{\mathrm{switch}}$. The penalty is included by a probability $p_{\mathrm{discard}} = \varepsilon\ell$ of discarding a configuration when a segment is in the same spot as a previous segment. If there are τ segments at a given site, then the probability of surviving this check is $(1 - p_{\mathrm{discard}})^{\tau} \approx \exp[-p_{\mathrm{discard}}\tau]$.

For our simulations, we modified the algorithm described by Smithe *et al.*[8] to include correlations between steps. In this algorithm, a fixed number of chains grow in parallel. Each chain is grown by taking steps on a one-dimensional lattice. If step number $j-1$ is in the positive (negative) direction, then step number j is taken in the same direction with probability $1-p_{\mathrm{switch}}$, and in the opposite direction with probability p_{switch}. If the step lands on a site that it has already visited τ times, the chain growth continues to step $j+1$ with probability $\exp(-p_{\mathrm{discard}}\tau)$, and is discarded with probability $1 - \exp(-p_{\mathrm{discard}}\tau)$. If a chain is discarded, then one of the other chains is duplicated in order to keep the number of chains constant. Details of how to calculate weighted averages from this algorithm are described elsewhere.[8,9] To obtain the master curve for the mean and variance of the extension shown in Fig. 4 in the main text we performed simulations of the telegraph model, using the algorithm

described above. For these simulations we set p_{discard} to 0.1, 0.01, or 0.001, and varied p_{switch} in logarithmic steps from 2^{-1} to 2^{-11}.

Now consider how to determine the scaling parameter α for the discretized process. Recalling that $v_0 = 1$, we have

$$\alpha \equiv \frac{\varepsilon}{2v_0 r} = \frac{1}{2}\frac{p_{\text{discard}}}{p_{\text{switch}}}\,. \tag{S-17}$$

However, the discretized approximation of the telegraph process is equivalent to the continuous process only when $p_{\text{switch}} \ll 1$, if α is not small. When α is small, on the other hand, then this constraint does not matter because we know that the details of the microscopic statistics are unimportant for the macroscopic statistics in this limit (see main text), provided that the effective step sizes (Kuhn lengths) of the continuous and discrete processes match so that the excluded volume per step is the same. For the continuous process with $v_0 = 1$ the effective step size is

$$\int_{-\infty}^{\infty} \mathrm{d}t\, \exp(-2r|t|) = r^{-1}\,. \tag{S-18}$$

For the discretized telegraph model that we simulate, the effective step size equals

$$\ell \sum_{n=-\infty}^{\infty} C(n) = \ell(p_{\text{switch}}^{-1} - 1)\,. \tag{S-19}$$

Here $C(n) \equiv (1 - 2p_{\text{switch}})^{|n|}$ is the correlation function of the discretized telegraph process. Replacing r^{-1} in Eq. (S-17) by the effective step size (S-19), we find that in the discretized model the scaling parameter must be computed as:

$$\alpha = \frac{p_{\text{discard}}}{2}\frac{1 - p_{\text{switch}}}{p_{\text{switch}}}\,. \tag{S-20}$$

In the limit $p_{\text{switch}} \to 0$ we obtain Eq. (S-17), as expected. In the opposite limit, $p_{\text{switch}} \to 1/2$, we obtain $\alpha \to p_{\text{discard}}/2$. For weak self avoidance ($p_{\text{discard}} \ll 1$) this random-walk limit corresponds to small values of α, so that the discretized process is an accurate approximation

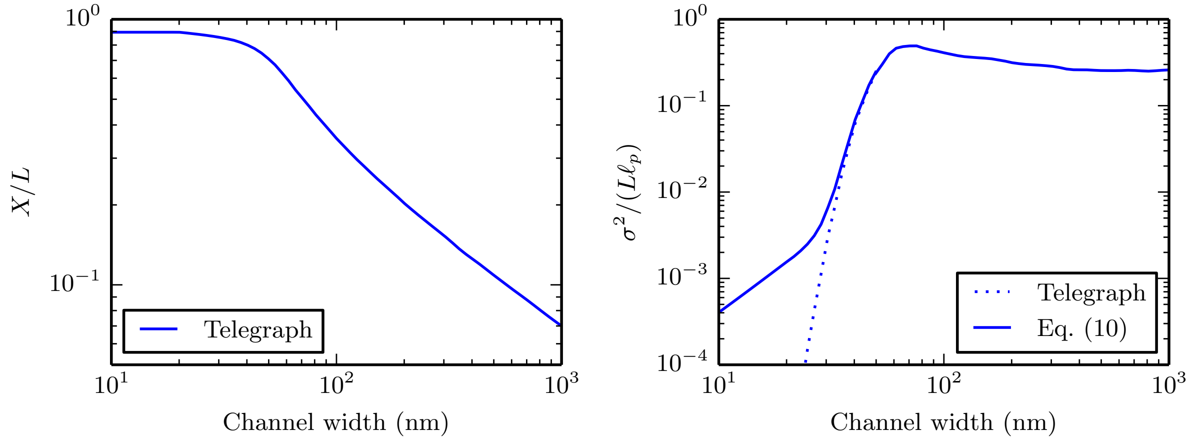

Figure S-2: Results of telegraph-model simulations for $\ell_{\mathrm{P}} = 50$ nm and $w = 5$ nm as a function of the channel width for the fractional extension (left) and for the normalized variance (right).

in this limit, if we use Eq. (S-20).

We ensure by inspection that the large-T limit was reached for the included simulations. Further, for all simulations included in the analysis, the total time T obeys $2Tr > 30$.

Results of our computer simulations of the telegraph model are summarized in Fig. 4 in the main text, showing scaled extension and extension variance as a function of the scaling variable α. These scaled plots demonstrate the universal nature of the telegraph-model predictions, as they collapse the data to a universal curve. However, for direct comparison of the telegraph-model predictions to experimental data on nano-confined DNA it may be practical to have the telegraph-model results in unscaled, dimensional form. The conversion between dimensional and dimensionless variables is readily obtained from Fig. 3 in the main text. Fig. S-2 shows the result for typical values for DNA in a buffer of high ionic strength: $\ell_{\mathrm{P}} = 50$ nm and $w = 5$ nm. The results are plotted as a function of the channel width D.

S-2 Ideal-polymer simulations

The data in Fig. 3 in the main text were obtained from simulations of confined *ideal* wormlike chains using the algorithm of Dai *et al.*[9] The chain consists of N_{b} touching beads with diameter d, so that the bond length is $a_{\mathrm{b}} = d$. All other lengths are expressed in terms of

the bond length a_b. The semiflexibility of the chain is imposed through a bending energy

$$U_\mathrm{bend} = k_B T \kappa (1 - \cos\varphi), \tag{S-21}$$

based on the angle φ between contiguous trios of beads. The prefactor for the bending energy, κ, is related to the persistence length ℓ_P and bond length a_b as[10]

$$\frac{\ell_\mathrm{P}}{a_\mathrm{b}} = \frac{\kappa}{\kappa - \kappa \coth(\kappa) + 1}\,, \tag{S-22}$$

The alignment parameter a and the global persistence length g were calculated by fitting the simulated correlation function to Eq. (2) in the main text. The parameter ε was computed by evaluating the average in Eq. (S-11) using the ideal wormlike chain simulations.

S-2.1 Ideal tangent correlations. Determination of g and a in terms of D and ℓ_P

Eq. (2) of the main text defines the parameters a and g in terms of the y-intercept and decay length, respectively, of the exponentially decaying correlation function of the confined *ideal* polymer, disregarding self avoidance. We extract these parameters from ideal-polymer simulations by directly measuring the correlation function $C(t)$ of the ideal polymer, for segments far from either end of the polymer. Some example measurements are shown in Fig. S-3, together with the result of fitting the correlation function to an exponential function. Note that in the right panel for very short separations $t < \lambda$, the decay is not exponential. Otherwise the agreement is excellent. The resulting values of a and g are plotted versus D/ℓ_P in Fig. S-4 (a) and (b). The simulations were performed by varying D as well as ℓ_P. The parameters a and g/ℓ_P are functions of D/ℓ_P only, since changing D and ℓ_P by the same factor has no physical significance, but is equivalent to a corresponding change in the unit of length.

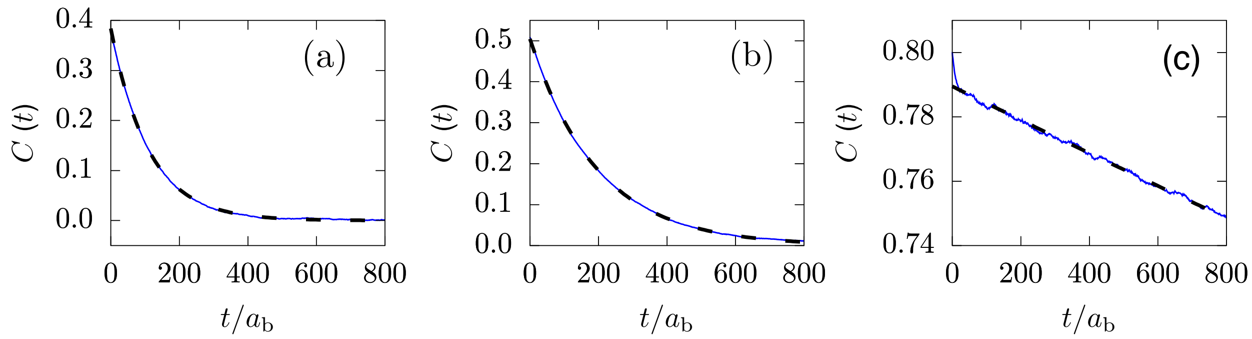

Figure S-3: Tangent correlation function measured in ideal simulations described in Section S-2 (solid blue line) and fitted to an exponential function (dashed black line). a) Wide channel ($D = 4\ell_P$). b) Intermediate channel ($D = 1.6\ell_P$). c) Narrow channel ($D = 0.43\ell_P$).

S-2.2 Determination of the parameter ε in terms of D, w, ℓ_P

The parameter ε is obtained by evaluating in confined ideal-polymer simulations the average in Eq. (S-11). To this end, we discretize the delta function by dividing the channel cross-section into $N_{\text{bins}} \times N_{\text{bins}}$ bins of size $\mathrm{d}y \times \mathrm{d}z$. The value of the discretized delta function is $(\mathrm{d}y\mathrm{d}z)^{-1}$ when $\boldsymbol{x}_\perp$ and $\boldsymbol{x}'_\perp$ are in the same bin, and zero otherwise. To approximate the average in Eq. (S-11), we increment a counter c by $\sin(\theta)/(\mathrm{d}y\mathrm{d}z)$ whenever two segments are in the same bin. Our estimate of ε is then given by $c \times 2w/N_{\text{iter}}$, where N_{iter} is the number of iterations in the simulations.

Note that a correct computation of the average requires that the two segments are well separated from both each other, and from either end of the polymer. Here, well separated means that the contour separation should be large enough that the two positions are statistically independent. This is the case if the contour length separation is much larger than $D^{2/3}\ell_P^{1/3} + D^2/\ell_P$.[11] To ensure that this is indeed the case in simulations, the contour length between the two segments was kept at either 30 or 60 times this limit. To avoid end effects, we further required that both segments in the pair were located at least a contour separation of $L/3$ from the left and right end, respectively.

Further, the number of bins in the discretization of the average must be large enough that the discretization error is negligible. In simulations we divided the channel cross section into 40×40, 60×60, or 80×80 bins. No significant difference was observed.

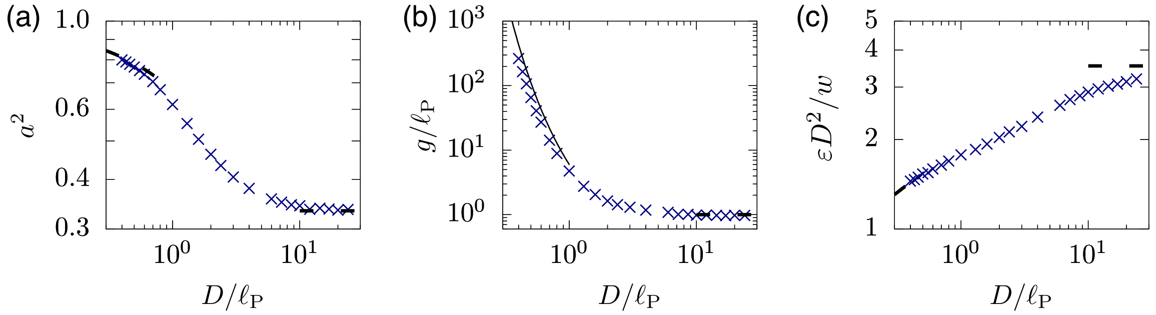

Figure S-4: Dependence of a, g, and ε upon D/ℓ_P. Results of ideal-polymer simulation as described in the text. The solid line is the result from Muralidhar *et al.*[12] The dashed lines represent different asymptotic limits discussed in this Supplemental Material. Fitting the constant C in Eq. (S-15) to the ideal-polymer simulations gives $C \approx 1.95$.

Finally, to avoid discretization error in the polymer configuration, the bead diameter d must be significantly smaller than the channel width D. In simulations we ensured that $D > 40d$. Again, we observed no effect from changing D/d. Fig. S-4(c) shows the result for ε as a function of D/ℓ_P, computed from these ideal-polymer simulations (symbols). To obtain Fig. S-4(c), D and ℓ_P were varied. A similar argument as in the previous Section shows that $\varepsilon D^2/w$ is a function of D/ℓ_P only.

S-2.3 Known asymptotes of g, a and p for small and large D/ℓ_P

Now we discuss the asymptotes shown in Fig. S-4. In panel (a) the dashed line for small values of D/ℓ_P is the Odijk result for X^2/L^2 as computed by Burkhardt *et al.*[13]. In panel (c) the dashed line for small values of D/ℓ_P is the prediction in the backfolded Odijk regime.[7,12] The dashed lines for large values of D/ℓ_P are the predictions for the extended de Gennes regime,[6] as given in Section S-1.3. For the global persistence length, we also compared our results to the previous simulations by Muralidhar *et al.*,[12] which were obtained using a different method, and a slightly different definition of g, solid line in Fig. S-4(b). We find good agreement.

S-3 Direct numerical simulations of the three-dimensional self-avoiding polymer in confinement

We have thus far explained how we simulated the telegraph model (in §S-1.4) and how we computed the parameter α from ideal chain simulations (in §S-2). We now proceed to describe the most computationally expensive simulations, namely the direct numerical simulation of a wormlike chain confined in a nanochannel with excluded volume. The direct numerical simulation (DNS) data in Fig. 4 in the main text were obtained by simulating a confined *self-avoiding* discrete wormlike chain via the Pruned Enriched Rosenbluth Method (PERM)[14,15]. The polymer model is similar to the one used for the simulations of ideal polymers in Section S-2, with the important difference that we now take self avoidance into account. We have previously developed highly efficient simulation methods for this problem[16,17] that we modified here to more accurately reflect the cylindrical excluded volume.

The bending energy is given by Eq. (S-21), and the persistence length is determined by Eq. (S-22). As in Section S-2, the chain consists of $N_{\rm b}$ touching beads of diameter d. Bead-bead excluded volume is treated as an infinite penalty if the center-to-center distance between the beads is less their diameter (representing the effective width w of the polymer). To approximate the cylindrical excluded volume interactions while retaining good resolution of the bending of the chain, we use $d/a_{\rm b} = 2$, rather than the customary $d/a_{\rm b} = 1$. The overlap penalty was not included for contiguous beads along the backbone that would overlap due to the choice $d > a_{\rm b}$. A hard-core excluded volume penalty is imposed also for bead-wall overlap, such that the beads are confined inside a channel of size $D_{\rm eff} = D - w$. For a given parameter set $(\ell_{\rm P}, D)$, we grew chains using an off-lattice PERM algorithm for confined polymers[16,18] to $N_{\rm b}$ = 40,000 beads, using 10 independent simulations with 100,000 tours per simulation. The values of X and σ^2 represent the average over these 10 sets, and we use the standard error of the mean computed from these independent sets to estimate the sampling error. In many cases, the error is smaller than the symbol size. For each pair $(\ell_{\rm P}, D)$, we confirmed that both X and σ^2 achieved linear scaling with L. Table S-1 lists all combinations of the persistence length and channel width used to produce the data in Fig. 4

of the main text.

Table S-1: List of parameters for PERM simulations.

ℓ_{P}	w	D
24.00	2	11.6
24.00	2	12.32
24.00	2	13.04
24.00	2	14
24.00	2	15.2
24.00	2	16.4
24.00	2	18.8
24.00	2	21.2
24.00	2	26
24.00	2	32
24.00	2	40.4
24.00	2	50
24.00	2	59.6
24.00	2	74
24.00	2	98
24.00	2	146
48.00	2	21.2
48.00	2	22.64
48.00	2	24.08
48.00	2	26
48.00	2	28.4
48.00	2	30.8
48.00	2	35.6
48.00	2	40.4
48.00	2	50
48.00	2	62
48.00	2	78.8
48.00	2	98
48.00	2	117.2
48.00	2	146
48.00	2	194
48.00	2	290
72.00	2	30.8
72.00	2	32.96
72.00	2	35.12
72.00	2	38
72.00	2	41.6
72.00	2	45.2

Continued on next page

Table S-1 – continued from previous page

ℓ_P	w	D
72.00	2	52.4
72.00	2	59.6
72.00	2	74
72.00	2	92
72.00	2	117.2
72.00	2	146
72.00	2	174.8
72.00	2	218
72.00	2	290
72.00	2	434
18.95	2	18.23
9.34	2	14.99
12.27	2	21.46
18.90	2	36.36
24.55	2	49.24
6.17	2	16.34
9.35	2	30.02
12.32	2	43.14
18.97	2	73.02
24.50	2	98.23
7.78	2	23.24
9.45	2	30.45
12.91	2	45.76
16.79	2	63.18
15.02	2	68.97
4.95	2	14.09
9.79	2	12.78
9.79	2	21.55
9.79	2	57.99
9.79	2	70.28
11.13	2	33.47
11.13	2	33.67
11.13	2	33.95
11.13	2	34.47
6.03	2	12.03
6.03	2	12.15
11.13	2	28.78
11.13	2	37.36
13.60	2	46.29
13.60	2	52.91
21.75	2	43.88
21.75	2	104.85

Continued on next page

Table S-1 – continued from previous page

ℓ_P	w	D
21.75	2	128.47
7.35	2	34.65
7.35	2	37.03
7.35	2	39.25
5.95	2	23.21
11.26	2	57.88
16.99	2	96.48
22.37	2	133.12
16.99	2	43.08
18.21	2	42.59
11.26	2	23.50
7.21	2	12.68

S-4 Summary of experimental data analyzed in main text

Here we explain how the experimental data sets in Figs. 1 and 4 in the main text were selected, and how the effective channel width, persistence length ℓ_P, and effective w were computed from the experimental parameters.

S-4.1 Computation of contour length, persistence length and effective polymer width

All the selected experiments presented in Figs. 1 and 4 in the main text used bis-intercalating dyes (TOTO-1 or YOYO-1) for visualization of DNA. These dyes are expected to increase the contour length, L linearly in proportion to the amount of dye bound. In order to calculate L, we assume that every bound dye molecule adds 0.44 nm to the bare contour length of 0.34 nm per base pair.[19] Note that the intercalation effect was neglected for Reinhart *et al.*,[20] who used a very low dye ratio (1 dye molecule per 40 base pairs).

There has been a substantial debate about the effect of bis-intercalating dyes on persistence length of DNA, ℓ_P, as summarized by Kundukad *et al.*[21] We concur with their conclusion that the persistence length is at best weakly affected by YOYO if the system is equilibrated. Therefore, we only account for the effect of ionic strength of DNA solutions when computing the persistence length. The ionic strength for the various buffers used in these experiments were calculated using a MATLAB program developed in our previous work.[19] Thereafter, ℓ_P was calculated using Dobrynin's empirical formula[22] which has been experimentally validated.[23] The effective polymer width w was computed from Stigter's theory.[24]

S-4.2 Tabulation of experimental data

Fig. 1 in the main text reports the parameters from experiments for cases where the aspect ratio is no greater than 1.5 and ℓ_P/w is no less than 2 to ensure (i) that the channels are close to square, in order to correspond to our simulations, and (ii) that the DNA can be modeled as semiflexible chains. The raw data appearing in that Figure are tabulated in Table S-2.

The effective channel width is computed by

$$D_{\text{eff}} = \sqrt{(D_1 - w)(D_2 - w)} \quad \text{(S-23)}$$

where D_1 and D_2 are the two dimensions of the channel and w is an approximation for the excluded volume due to DNA-wall electrostatic interactions.

Table S-2: Experiments in Fig. 1 of the main text.

Source	Ionic Strength (mM)	ℓ_P (nm)	w (nm)	D_1 (nm)	D_2 (nm)
Reisner *et al.*[25]	16	61	13	30	40
	16	61	13	60	80
	16	61	13	80	80
	16	61	13	130	140
	16	61	13	300	440
	16	61	13	440	440
Reisner *et al.*[26]	4	76	25	50	50
	14	62	13	50	50
	30	57	9	50	50
	107	52	5	50	50
	4	76	25	100	100
	14	62	13	100	100
	30	57	9	100	100
	106	52	6	100	100
	261	50	4	100	100
	4	75	24	200	200
	14	62	13	200	200
	30	57	9	200	200
	107	52	5	200	200
	259	50	4	200	200
	8	67	17	200	200
	14	62	13	200	200
	35	56	9	200	200
	74	53	6	200	200
Thamdrup *et al.*[27]	53	54	7	250	250

Continued on next page

Table S-2 – continued from previous page

Source	Ionic Strength (mM)	ℓ_P (nm)	w (nm)	D_1 (nm)	D_2 (nm)
Zhang *et al.*[28]	1	106	53	200	300
	2	89	36	200	300
	3	83	31	200	300
	10	65	15	200	300
	27	58	10	200	300
	33	57	9	200	300
	1	106	53	300	300
	2	89	36	300	300
	3	83	31	300	300
	11	65	15	300	300
	27	58	10	300	300
	33	57	9	300	300
Utko *et al.*[29]	23	59	11	150	208
	23	59	11	150	221
Kim *et al.*[30]	1	103	50	250	250
	2	88	35	250	250
Werner *et al.*[31]	4	76	25	180	121
	4	76	25	180	259
	23	59	11	180	128
	23	59	11	180	262
	40	56	8	180	123
	40	56	8	180	261
Gupta *et al.*[19]	7	69	19	100	100
Reinhart *et al.*[20]	103	52	6	40	40
	103	52	6	42	42
	103	52	6	43	43
	103	52	6	49	49
	103	52	6	51	51
Gupta *et al.*[32]	7	69	19	300	350
	7	69	19	300	450
Iarko *et al.*[33]	4	77	26	300	302
	24	59	10	300	302
	77	53	6	300	302
	184	51	5	300	302
	4	77	26	120	150

Continued on next page

Table S-2 – continued from previous page

Source	Ionic Strength (mM)	ℓ_P (nm)	w (nm)	D_1 (nm)	D_2 (nm)
	77	53	6	120	151
Alizadehheidari *et al.*[34]	95	52	6	100	150
	24	59	10	100	150
	7	69	19	100	150
	4	77	26	100	100

Fig. 4 in the main text reports values of X and σ^2 from experiments for cases where the aspect ratio is no greater than 1.5 and ℓ_P/w is no less than 2. Table S-3 provides a tabulated list of the experimental parameters and data.

Table S-3: Experimental data in Fig. 4 of the main text. The values of α marked with an asterisk are obtained by extrapolation.

Source	ℓ_P/w	D_{eff}/ℓ_P	α	X/L	$\sigma^2/(L\ell_P)$	L (μm)
Reisner *et al.*[25]	4.9	0.4	1543*	0.73		18.6
	4.9	0.9	3.05	0.63		18.6
	4.9	1.1	1.50	0.50		18.6
	4.9	2.0	0.25	0.36		18.6
	4.9	5.7	0.03	0.15		18.6
	4.9	7.0	0.02	0.12		18.6
Reisner *et al.*[26]	3.1	0.3	5119*	0.82		21.0
	4.7	0.6	32.9	0.82		21.0
	6.1	0.7	8.44	0.74		21.0
	9.5	0.9	2.20	0.60		21.0
	3.1	1.0	3.59	0.73		21.0
	4.7	1.4	0.70	0.55		21.0
	6.1	1.6	0.36	0.45		21.0
	9.5	1.8	0.16	0.60		21.0
	12.3	1.9	0.11	0.32		21.0
	3.1	2.3	0.28	0.45		21.0
	4.7	3.0	0.11	0.26		21.0
	6.2	3.3	0.07	0.23		21.0
	9.5	3.7	0.03	0.17		21.0
	12.2	3.9	0.02	0.15		21.0
	3.9	2.7	0.16	0.26		21.0
	4.7	3.0	0.11	0.27		21.0
	6.5	3.4	0.06	0.20		21.0
	8.4	3.6	0.04	0.16		21.0
Thamdrup *et al.*[27]	7.5	4.5	0.03	0.19	0.26	70.9
Zhang *et al.*[28]	2.0	1.8	0.79	0.23		59.5
	2.4	2.3	0.34	0.21		59.5
	2.6	2.6	0.27	0.19		59.5

Continued on next page

Table S-3 – continued from previous page

Source	ℓ_P/w	D_{eff}/ℓ_P	α	X/L	$\sigma^2/(L\ell_P)$	L (μm)
	4.2	3.5	0.09	0.19		59.5
	5.9	4.1	0.05	0.17		59.5
	6.3	4.2	0.04	0.19		59.5
	2.0	2.3	0.43	0.19		59.5
	2.4	3.0	0.21	0.17		59.5
	2.7	3.2	0.16	0.17		59.5
	4.2	4.4	0.06	0.16		59.5
	5.9	5.0	0.03	0.14		59.5
	6.3	5.1	0.03	0.15		59.5
Utko *et al.*[29]	5.6	2.8	0.10	0.31		18.6
	5.6	2.8	0.10	0.29		18.6
	5.6	2.9	0.10	0.31		18.6
	5.6	2.9	0.10	0.31		18.6
Kim *et al.*[30]	2.1	1.9	0.64	0.62		21.8
	2.5	2.4	0.31	0.57		21.8
Werner *et al.*[31]	3.0	1.6	0.73	0.53		18.6
	3.0	1.6	0.71	0.47		18.6
	3.0	1.7	0.65	0.48		18.6
	3.0	1.6	0.67	0.50		18.6
	3.0	1.7	0.63	0.51		18.6
	3.0	1.7	0.59	0.48		18.6
	3.0	1.8	0.56	0.47		18.6
	3.0	1.8	0.53	0.45		18.6
	3.0	1.8	0.50	0.46		18.6
	3.0	1.8	0.51	0.44		18.6
	3.0	1.9	0.47	0.44		18.6
	3.0	1.9	0.47	0.46		18.6
	3.0	1.9	0.45	0.44		18.6
	3.0	2.0	0.42	0.42		18.6
	3.0	2.0	0.39	0.41		18.6
	3.0	2.1	0.38	0.39		18.6
	3.0	2.1	0.37	0.41		18.6
	3.0	2.1	0.35	0.41		18.6
	3.0	2.2	0.34	0.39		18.6
	3.0	2.2	0.32	0.40		18.6
	3.0	2.2	0.31	0.38		18.6
	3.0	2.3	0.30	0.39		18.6
	3.0	2.3	0.29	0.37		18.6

Continued on next page

Table S-3 – continued from previous page

Source	ℓ_P/w	D_{eff}/ℓ_P	α	X/L	$\sigma^2/(L\ell_P)$	L (μm)
	3.0	2.4	0.27	0.38		18.6
	3.0	2.4	0.27	0.37		18.6
	3.0	2.4	0.26	0.35		18.6
	3.0	2.4	0.25	0.37		18.6
	3.0	2.5	0.25	0.34		18.6
	3.0	2.4	0.26	0.34		18.6
	5.6	2.4	0.14	0.31		18.6
	5.6	2.5	0.14	0.30		18.6
	5.6	2.5	0.13	0.32		18.6
	5.6	2.5	0.13	0.31		18.6
	5.6	2.6	0.12	0.31		18.6
	5.6	2.7	0.11	0.29		18.6
	5.6	2.8	0.10	0.28		18.6
	5.6	2.9	0.10	0.28		18.6
	5.6	3.0	0.09	0.28		18.6
	5.6	3.1	0.09	0.26		18.6
	5.6	3.1	0.09	0.25		18.6
	5.6	3.2	0.08	0.28		18.6
	5.6	3.2	0.08	0.27		18.6
	5.6	3.3	0.07	0.26		18.6
	5.6	3.4	0.07	0.24		18.6
	5.6	3.5	0.07	0.25		18.6
	5.6	3.5	0.07	0.24		18.6
	6.8	2.5	0.11	0.29		18.6
	6.8	2.6	0.10	0.28		18.6
	6.8	2.6	0.10	0.26		18.6
	6.8	2.8	0.09	0.27		18.6
	6.8	2.8	0.08	0.26		18.6
	6.8	3.0	0.08	0.26		18.6
	6.8	3.1	0.07	0.25		18.6
	6.8	3.1	0.07	0.23		18.6
	6.8	3.3	0.06	0.24		18.6
	6.8	3.3	0.06	0.24		18.6
	6.8	3.4	0.06	0.23		18.6
	6.8	3.8	0.05	0.21		18.6
	6.8	3.6	0.05	0.22		18.6
	6.8	3.7	0.05	0.21		18.6
Gupta *et al.*[19]	3.7	1.2	1.53	0.81	0.21	18.6
Reinhart *et al.*[20]	9.3	0.66	8.40	0.89	0.03	

Continued on next page

Table S-3 – continued from previous page

Source	ℓ_P/w	D_{eff}/ℓ_P	α	X/L	$\sigma^2/(L\ell_P)$	L (μm)
	9.3	0.70	6.15	0.88	0.02	
	9.3	0.72	5.32	0.87	0.04	
	9.3	0.83	2.53	0.84	0.09	
	9.3	0.87	2.06	0.81	0.13	
Gupta *et al.*[32]	3.7	4.4	0.06	0.28	0.26	63.6
	3.7	4.8	0.06	0.26	0.27	63.6
	3.7	5.1	0.05	0.25	0.27	63.6
Iarko *et al.*[33]	3.0	3.6	0.12	0.27	0.24	63.6
	5.6	5.0	0.03	0.22	0.32	62.4
	8.5	5.6	0.02	0.18	0.31	60.9
	11.2	5.9	0.01	0.16	0.33	58.9
	3.0	1.4	1.08	0.58	0.25	63.6
	8.5	2.4	0.09	0.31	0.45	57.9
Alizadehheidari *et al.*[34]	9.1	2.2	0.10		0.29	16.2
	5.6	1.9	0.24		0.23	16.2
	3.6	1.5	0.75		0.19	16.2
	3.0	1.0	4.24		0.12	16.2

S-4.3 Experimental parameters in relation to asymptotic scaling regimes

In the main text, we claim that the disagreement noted earlier between experiments and the scalings $X \sim D^{-2/3}$ or $X \sim \xi^{1/3}$ reflects that the DNA experiments do not satisfy the strong inequalities $D \gg \ell_P$ or $D \ll \ell_P$. To support this claim, Fig. S-5 reproduces the data in Fig. 1 of the main text but also indicates different asymptotic scaling regimes:[7] the Odijk regime; backfolded Odijk (BFO) regime; extended de Gennes (EDG) regime; and de Gennes regime. The condition for the boundary between the Odijk regime and the BFO regime is[7,12] $\xi = 1$, corresponding to Eq. (S-16) with $C = 1$. The boundary between the BFO and EDG regimes is[7] $D_{\text{eff}} = 2\ell_P$. The boundary between the EDG and the de Gennes regimes is $D_{\text{eff}} = \ell_P^2/w$, as stated in the main text. Fig. S-5 demonstrates that most recent experiments on nanoconfined DNA are performed close to these boundaries separating the different asymptotic regimes. Near the boundaries, that is for most experiments shown in

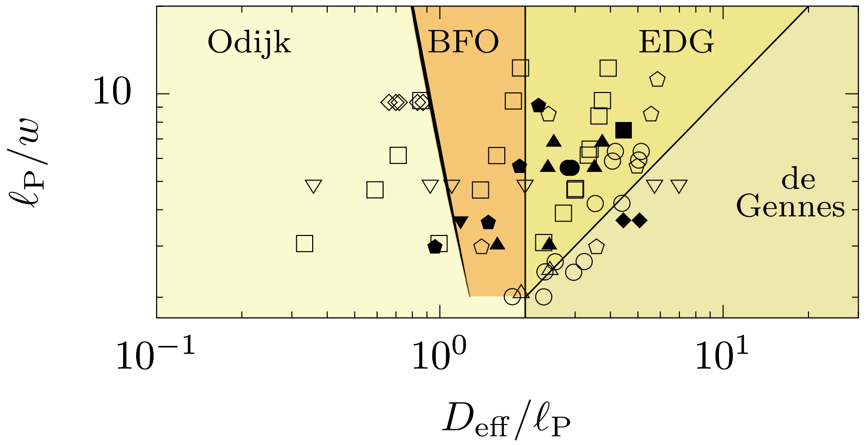

Figure S-5: Same data as Fig. 1 of the main text, here with the boundaries between the different asymptotic regimes that are mentioned in Section S-4.3, solid lines.

Fig. S-5, existing scaling laws must fail to describe the extension of DNA in a nanochannel.

References

[1] Odijk, T. On the Statistics and Dynamics of Confined or Entangled Stiff Polymers. *Macromolecules* **16**, 1340–1344 (1983).

[2] van der Hofstad, R., den Hollander, F. & König, W. Weak interaction limits for one-dimensional random polymers. *Probability Theory and Related Fields* **125**, 483–521 (2003).

[3] Grosberg, A. Y. & Khokhlov, A. R. *Statistical Physics of Macromolecules* (AIP press, 1994).

[4] Werner, E., Westerlund, F., Tegenfeldt, J. O. & Mehlig, B. Monomer Distributions and Intrachain Collisions of a Polymer Confined to a Channel. *Macromolecules* **46**, 6644–6650 (2013).

[5] Onsager, L. The effects of shape on the interaction of colloidal particles. *Ann. N. Y. Acad. Sci.* **51**, 627–659 (1949).

[6] Werner, E. & Mehlig, B. Confined polymers in the extended de Gennes regime. *Phys. Rev. E* **90**, 062602 (2014).

[7] Odijk, T. Scaling theory of DNA confined in nanochannels and nanoslits. *Phys. Rev. E* **77**, 060901(R) (2008).

[8] Smithe, T. S. C., Iarko, V., Muralidhar, A., Werner, E., Dorfman, K. D. & Mehlig, B. Finite-size corrections for confined polymers in the extended de Gennes regime. *Phys. Rev. E* **92**, 062601 (2015).

[9] Dai, L., van der Maarel, J. R. C. & Doyle, P. S. Extended de Gennes regime of DNA confined in a nanochannel. *Macromolecules* **47**, 2445–2450 (2014).

[10] Muralidhar, A. & Dorfman, K. D. Kirkwood diffusivity of long semiflexible chains in nanochannel confinement. *Macromolecules* **48**, 2829–2839 (2015).

[11] Chen, J. Z. Y. Free energy and extension of a wormlike chain in tube confinement. *Macromolecules* **46**, 9837–9844 (2013).

[12] Muralidhar, A., Tree, D. R. & Dorfman, K. D. Backfolding of wormlike chains confined in nanochannels. *Macromolecules* **47**, 8446–8458 (2014).

[13] Burkhardt, T. W., Yang, Y. & Gompper, G. Fluctuations of a long, semiflexible polymer in a narrow channel. *Phys. Rev. E* **82**, 041801 (2010).

[14] Grassberger, P. Pruned-enriched Rosenbluth method: Simulations of θ polymers of chain length up to 1 000 000. *Phys. Rev. E* **56**, 3682–3693 (1997).

[15] Prellberg, T. & Krawczyk, J. Flat histogram version of the pruned and enriched rosenbluth method. *Phys. Rev. Lett.* **92**, 120602 (2004).

[16] Tree, D. R., Wang, Y. & Dorfman, K. D. Extension of DNA in a nanochannel as a rod-to-coil transition. *Phys. Rev. Lett.* **110**, 208103 (2013).

[17] Tree, D. R., Muralidhar, A., Doyle, P. S. & Dorfman, K. D. Is DNA a good model polymer? *Macromolecules* **46**, 8369–8382 (2013).

[18] Muralidhar, A. *Equilibrium properties of DNA and other semiflexible polymers confined in nanochannels*. Ph.D. thesis, University of Minnesota (2016).

[19] Gupta, D., Sheats, J., Muralidhar, A., Miller, J. J., Huang, D. E., Mahshid, S., Dorfman, K. D. & Reisner, W. Mixed confinement regimes during equilibrium confinement spectroscopy of DNA. *J. Chem. Phys.* **140**, 214901 (2014).

[20] Reinhart, W. F., Reifenberger, J. G., Gupta, D., Muralidhar, A., Sheats, J., Cao, H. & Dorfman, K. D. Distribution of distances between DNA barcode labels in nanochannels close to the persistence length. *J. Chem. Phys.* **142**, 064902 (2015).

[21] Kundukad, B., Yan, J. & Doyle, P. S. Effect of YOYO-1 on the mechanical properties of DNA. *Soft Matter* **10**, 9721–9728 (2014).

[22] Dobrynin, A. V. Effect of counterion condensation on rigidity of semiflexible polyelectrolytes. *Macromolecules* **39**, 9519–9527 (2006).

[23] Hsieh, C. C., Balducci, A. & Doyle, P. S. Ionic effects on the equilibrium dynamics of DNA confined in nanoslits. *Nano Lett.* **8**, 1683–1688 (2008).

[24] Stigter, D. Interactions of highly charged colloidal cylinders with applications to double-stranded DNA. *Biopolymers* **16**, 1435–1448 (1977).

[25] Reisner, W., Morton, K. J., Riehn, R., Wang, Y. M., Yu, Z., Rosen, M., Sturm, J. C., Chou, S. Y., Frey, E. & Austin, R. H. Statics and dynamics of single DNA molecules confined in nanochannels. *Phys. Rev. Lett.* **94**, 196101 (2005).

[26] Reisner, W., Beech, J. P., Larsen, N. B., Flyvbjerg, H., Kristensen, A. & Tegenfeldt, J. O. Nanoconfinement-enhanced conformational response of single DNA molecules to changes in ionic environment. *Phys. Rev. Lett.* **99**, 058302 (2007).

[27] Thamdrup, L. H., Klukowska, A. & Kristensen, A. Stretching DNA in polymer nanochannels fabricated by thermal imprint in PMMA. *Nanotechnology* **19**, 125301 (2008).

[28] Zhang, C., Zhang, F., Van Kan, J. A. & Van Der Maarel, J. R. C. Effects of electrostatic screening on the conformation of single DNA molecules confined in a nanochannel. *J. Chem. Phys.* **128**, 225109 (2008).

[29] Utko, P., Persson, F., Kristensen, A. & Larsen, N. B. Injection molded nanofluidic chips: fabrication method and functional tests using single-molecule DNA experiments. *Lab Chip* **11**, 303–308 (2011).

[30] Kim, Y., Kim, K. S., Kounovsky, K. L., Chang, R., Jung, G. Y., DePablo, J. J., Jo, K. & Schwartz, D. C. Nanochannel confinement: DNA stretch approaching full contour length. *Lab Chip* **11**, 1721–1729 (2011).

[31] Werner, E., Persson, F., Westerlund, F., Tegenfeldt, J. O. & Mehlig, B. Orientational correlations in confined DNA. *Phys. Rev. E* **86**, 041802 (2012).

[32] Gupta, D., Miller, J. J., Muralidhar, A., Mahshid, S., Reisner, W. & Dorfman, K. D. Experimental Evidence of Weak Excluded Volume Effects for Nanochannel Confined DNA. *ACS Macro Lett.* **4**, 759–763 (2015).

[33] Iarko, V., Werner, E., Nyberg, L. K., Müller, V., Fritzsche, J., Ambjörnsson, T., Beech, J. P., Tegenfeldt, J. O., Mehlig, K., Westerlund, F. & Mehlig, B. Extension of nanoconfined DNA: Quantitative comparison between experiment and theory. *Phys. Rev. E* **92**, 062701 (2015).

[34] Alizadehheidari, M., Werner, E., Noble, C., Reiter-Schad, M., Nyberg, L. K., Fritzsche, J., Mehlig, B., Tegenfeldt, J. O., Ambjornsson, T., Persson, F. & Westerlund, F. Nanoconfined circular and linear DNA: Equilibrium conformations and unfolding kinetics. *Macromolecules* **48**, 871–878 (2015).

[35] Tree, D. R., Wang, Y. & Dorfman, K. D. Modeling the relaxation time of DNA confined in a nanochannel. *Biomicrofluidics* **7**, 054118 (2013).
